# Supplementary material for: The Effect of Titanium Dioxide Surface Modification on the Dispersion, Morphology, and Mechanical Properties of Recycled PP/PET/TiO2 PBNANOs
Source: Polymers (Basel). 2019 Oct 16;11(10):1692. doi: 10.3390/polym11101692 (PMC6835408; doi:10.3390/polym11101692)
Supplement: Supplementary file 1 [file polymers-11-01692-s001.pdf]

The Effect of Titanium Dioxide Surface Modification on the Dispersion, Morphology, and Mechanical Properties of Recycled PP/PET/TiO_2 PBNANOs

Eider Matxinandiarena [1], Agurtzane Múgica [1], Manuela Zubitur [2], Cristina Yus [5], Víctor Sebastián [5,6], Silvia Irusta [5,6], Alfonso David Loaeza [7], Orlando Santana [7], Maria Lluisa Maspoch[7], Cristian Puig [4] and Alejandro J. Müller [1,3,*]

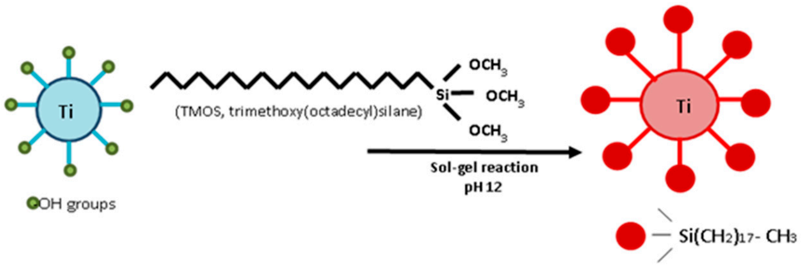

Figure S1: Hydrophilic nanoparticles functionalization scheme

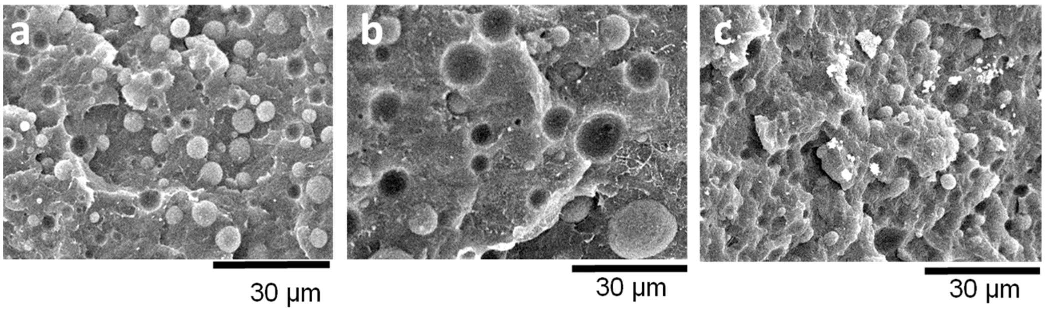

Figure S2. SEM image of PBNANO-hphi with a) 3% b) 7% c) 12%

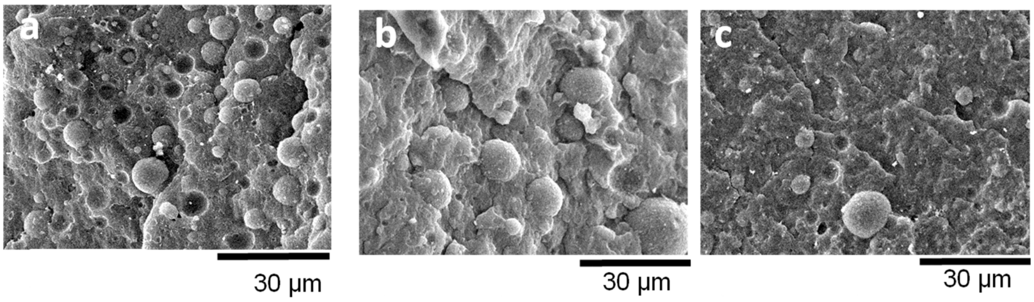

Figure S3. SEM image of PBNANO-hpho with a) 3% b) 7% c) 12%

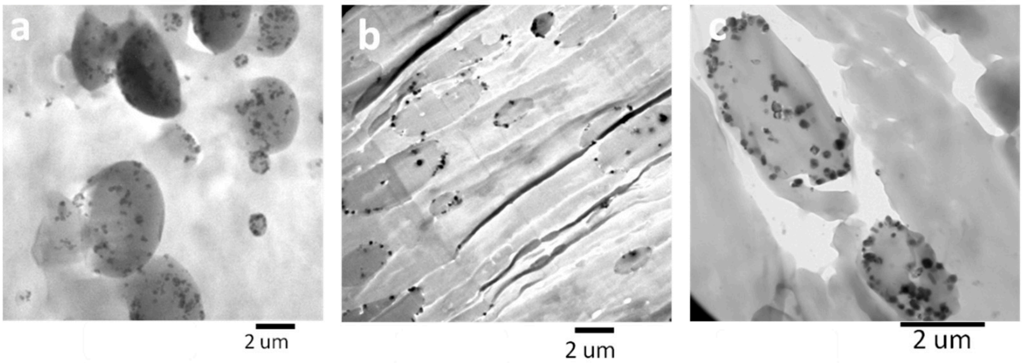

Figure S4. TEM image of PBNANO-hphi with a) 3% b) 7% c) 12%

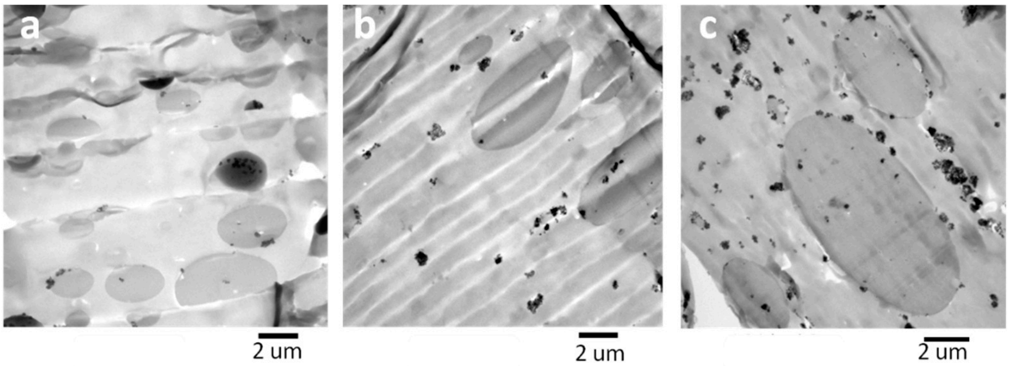

Figure S5. TEM image of PBNANO-hpho with a) 3% b) 7% c) 12%

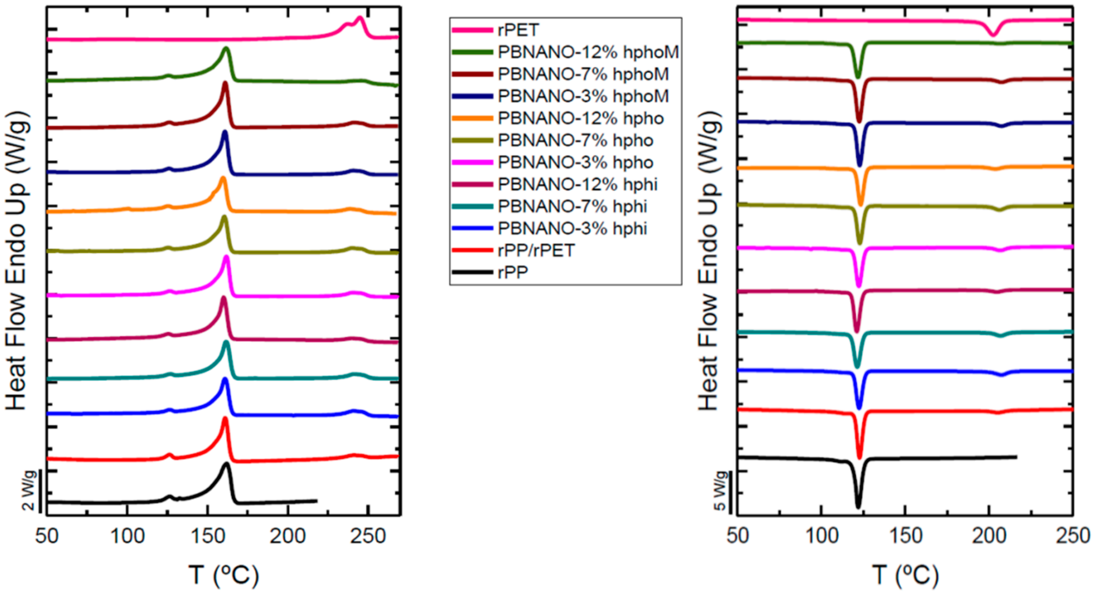

Figure S6. Nonisothermal DSC cooling scan down (left) and the subsequent heating (right)

Table A1. Calorimetric properties obtained from non-isothermal cooling DSC experiments

			$T_{c,PP}$ [°C]	$\Delta H_{c,PP}$ [J/g]	$T_{c,PET}$ [°C]	$\Delta H_{c,PET}$ [J/g]	$X_{c,PP}$ [°C]	$X_{c,PET}$ [°C]
**rPP**			125.5	93	-	-	45	-
**80rPP/20rPET**	**Total % hydrophilic TiO_2 (hphi)**	2	126.5	89	212.7	31	43	22
		3	126.3	80	213.2	56	39	40
		5	125.9	82	214.4	53	40	38
		7	125.3	81	212.9	49	39	35
		9.5	125.6	88	212.2	39	43	28
		12	124.6	94	210.8	33	45	24
	**Total % hydrophobic TiO_2 (hpho)**	3	125.7	81	212.3	44	39	31
		5	126.4	95	212.1	36	46	26
		7	126.2	80	212.7	53	38	38
		9.5	126.3	89	210.6	36	43	25
		12	126.8	85	210.1	38	41	27
	**Total % hydrophobically modified TiO_2 (hphoM)**	3	125.6	86	212.9	43	42	30
		5	125.5	89	213.5	44	43	33
		7	126.4	90	213.3	41	43	31
		9.5	122.5	94	200.9	24	46	17
		12	121.8	89	206.5	17	43	12
**rPET**			-	-	203.4	41	-	29

Table S2. Calorimetric properties obtained from non-isothermal heating DSC experiments

			$T_{m,PP}$ [°C]	$\Delta H_{m,PP}$ [J/g]	$T_{m,PET}$ [°C]	$\Delta H_{m,PET}$ [J/g]
**rPP**			162.0	78	-	-
**80rPP/20rPET**	**Total % hydrophilic TiO_2 (hphi)**	2	161.3	65	241.1	25
		3	161.1	73	241.5	49
		5	161.4	78	241.8	45
		7	161.7	76	241.3	41
		9.5	162.9	88	244.7	37
		12	160.0	96	240.4	26
	**Total % hydrophobic TiO_2 (hpho)**	3	162.1	80	241.6	36
		5	161.5	88	240.8	27
		7	161.3	75	240.9	46
		9.5	160.6	94	240.0	27
		12	160.4	90	240.1	34
	**Total % hydrophobically modified TiO_2 (hphoM)**	3	161.8	82	241.3	34
		5	161.2	83	241.4	35
		7	161.4	86	241.1	35
		9.5	160.3	92	243.7	23
		12	161.6	94	246.4	32

**rPET**	-	-	243.6	34

Table S3. Tensile mechanical parameters of the materials studied

Sample	Elastic Modulus [GPa]	Yielding strength [Mpa]	Yielding strain [%]	Strain at break [%]
rPET-O	2.20 ± 0.03	57.3 ± 0.5	3.1 ± 0.1	268 ± 13
rPP	1.07 ± 0.03	21.8 ± 0.2	4.9 ± 0.2	19 ± 4
PBNANO-0	1.26 ± 0.01	19.8 ± 0.5	4.2 ± 0.2	29 ± 2
PBNANO-hpho	1.42 ± 0.03	21.5 ± 0.5	3.2 ± 0.1	11 ±4
PBNANO-hphoM	1.33 ± 0.02	20.7 ± 0.3	2.5 ± 0.3	5.7 ± 0.3
PBNANO-hphi	1.34 ± 0.04	20.1 ± 0.3	4.2 ± 0.2	29 ± 5
